# Functional site prediction selects correct protein models

**DOI:** 10.1186/1471-2105-9-S1-S13

**Published:** 2008-02-13

**Authors:** Vijayalakshmi Chelliah, William R Taylor

**Affiliations:** 1 Division of Mathematical Biology, The National Institute for Medical Research, The Ridgeway, Mill Hill, London NW7 1AA, UK

## Abstract

**Background:**

The prediction of protein structure can be facilitated by the use of constraints based on a knowledge of functional sites. Without this information it is still possible to predict which residues are likely to be part of a functional site and this information can be used to select model structures from a variety of alternatives that would correspond to a functional protein.

**Results:**

Using a large collection of protein-like decoy models, a score was devised that selected those with predicted functional site residues that formed a cluster. When tested on a variety of small *α*/*β*/*α *type proteins, including enzymes and non-enzymes, those that corresponded to the native fold were ranked highly. This performance held also for a selection of larger *α*/*β*/*α *proteins that played no part in the development of the method.

**Conclusion:**

The use of predicted site positions provides a useful filter to discriminate native-like protein models from non-native models. The method can be applied to any collection of models and should provide a useful aid to all modelling methods from *ab initio *to homology based approaches.

## Background

The prediction of protein structure from purely sequence data has posed a challenge over many years. With the increasing numbers of known structures, many recent methods have turned to the use of structure-based sequence alignment (threading) [1,2] or fragment assembly [3], including various hybrid combinations [4]. Although some of these methods are referred to as *ab initio*, they all rely on having a database of known structures and are better classed as *de novo *to distinguish them from a pure physico-chemical approach.

Following some of the earliest attempts at protein structure prediction [5,6], it became clear that the use of external biochemical constraints on residue proximity could provide a very powerful filter on the permitted structures, whether these were simple pairwise positions [7] or whole motifs [8]. Biochemically important residues are typically found in close proximity and are also highly conserved. With a view to using such information to constrain predictions, attempts were made to predict active site residues from a multiple sequence alignment [9]. This approach relies on finding residues that are conserved for no apparent structural reason and some recent methods also combine this with the requirement to form a cluster in space when the protein structure (or a model) is known [10]. (See ref. [11], for a review).

In this work we use the method of Chelliah et al., (2004) [10] to predict residues that are likely to be located in an active (or binding) site and to evaluate their proximity in the context of a model for the protein. We do not address the generation of the models but rather take a series of 'decoy' models constructed by a *de novo *protein structure prediction method (See Methods section for details). Unlike some collections of decoy models, the ones we use were generated from abstract secondary structure lattice frameworks (Taylor, 2002) which has the advantage that we know, by definition, whether the model has the native fold. Rather than use an ambiguous Root Mean Square Deviation (RMSD) based measure, we can then evaluate our model scores by a true/false criterion.

We begin our study using a small sample of five *α*/*β*/*α *proteins for which a large number of decoys had been previously generated. These proteins, with length under 150 residues, are a mix of both topological and functional types, including enzymes and non-enzymes. We then develop a score termed "Fold Score" based on the proximity of predicted active (or other) site residues to rank the different folds on their functional potential. Without change, the method is then applied to a variety of other proteins of the same structural class but ranging in size up to almost 200 residues in length.

## Results and Discussion

### Training-set of five proteins

**Figure 1:**
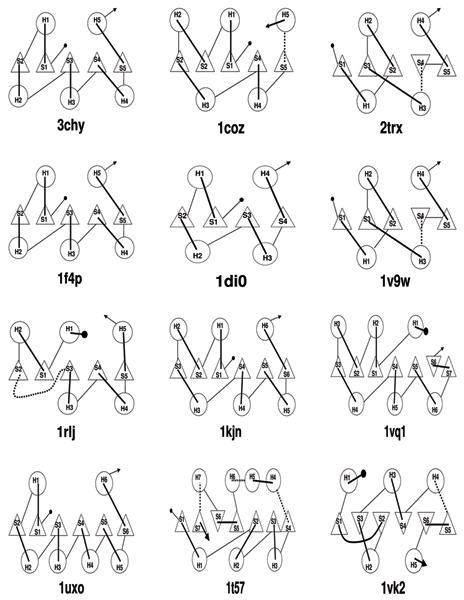
**Topology cartoons for the training-set and independent test-set proteins**. Each protein is shown with *β*-strands represented as triangles and α-helices as circles. They are identified by their PDB code. Training-set proteins: 3chy (Chemotaxis Y protein), 1coz (Glycerol-3-phosphate cytidylyltransferase), 1di0 (Lumazine synthase), 2trx (Thioredoxin), 1f4p (Flavodoxin). Independent test-set proteins: 1v9w, 1rlj, 1kjn, 1vq1, 1uxo, 1t57, 1vk2. Inverted triangle denotes the strands in the opposite direction.

**Figure 2:**
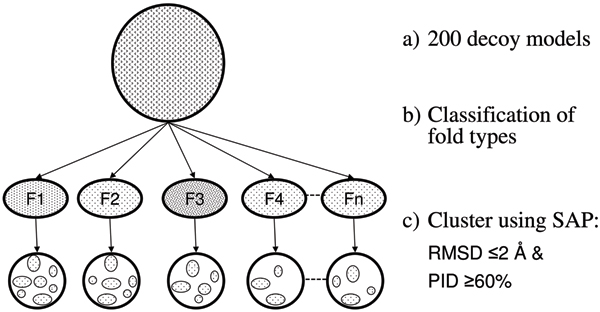
**Clustering of models**. Clustering of the 200 decoy models. (a) 200 decoy models obtained from the *de novo *protein structure prediction method. (b) Classification of the 200 models based on their fold-types. (c) Clustering of models of same fold-type by pair-wise superposition using SAP [18]. Models with ≤2 Å RMSD and ≥60% PID were clustered together.

**Figure 3:**
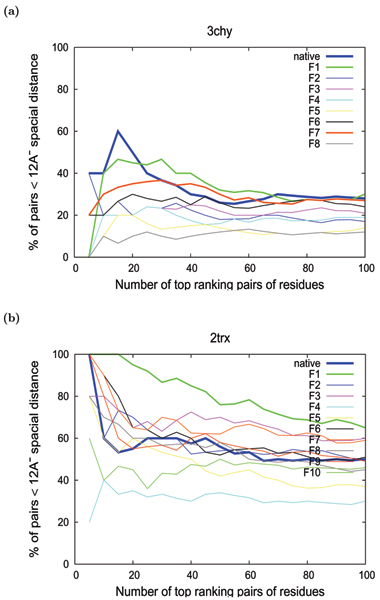
**Example "proximity plots" for **3chy**and **2trx. The "proximity plots" for the best models of each fold-type for (a) Chemotaxis Y protein (3chy) and (b) Thioredoxin protein (2trx) are shown. The thick blue line indicates the native crystal structure in both 3chy and 2trx plots. For 3chy, F1 (thick green line) and F7 (thick red line) corresponds to the correct fold. For 2trx, F1 (thick green line) corresponds to the correct fold.

The method was tested initially on a set of five small *β*/*α *proteins, with a central *β*-sheet packed either side by a layer of *α*-helix giving a three-layered *α*/*β*/*α *architecture (Figure 1). For each protein, 200 decoy models that contains folds of different types (topologies) were taken and were classified based on their fold-type and the models of same fold-type were clustered (Figure 2 and Methods section for details of the protein, decoy data and clustering of models). As an example, Table 1 gives the details of number of fold-types in the 200 models for one of these proteins with the others having a similar distribution. The scores calculated by the CRESCENDO method were analysed on each model by measuring the spatial proximity of different sized subsets of the highest scoring positions. A score (percentage of residue pairs that are less than 12 Å distance) was devised to measure how compact each subset was and plotted against increasing subset size. (See the Methods section for details). These "proximity plots" for the predicted site residues for each protein showed a clear overall trend for the smaller subsets of the most highly predicted residues to be in closer proximity. However, differences in loop conformation and strand or helix shifts can have major effect while looking at the proximity of the functional site residues. Even models with the same fold-type do not always have all their residues in the same spatial location and hence the "proximity plots" contains a degree of noise. Figures 3a and 3b shows the "proximity plots" of the best models in each fold-type for Chemotaxis Y protein (3chy) and Thioredoxin (2trx). When the sample of site residues was small (less than 5) there was more noise in the data and when the sample was large (over 50) the plots all decayed to an uninformative level of discrimination. For proteins that have multiple active sites, the top scoring pairs of residues could be from two different binding sites and may not be within the distance cut-off (12 Å). Some of the noise in the "proximity plots" (Figure 3a) for the smaller subsets (5 or 10 pairs of residues) is due to this reason. For example, CRESCENDO predicted two clusters of binding site residues for the Chemotaxis Y protein (3chy), one being the active site and other being the oligermic interface. The top scoring few pairs belong to both these two sites and were not in close proximity to each other. This is the reason for this protein having only 40% of the top 5 pairs of residues being within the 12 Å distance cut-off (Figure 3a). By contrast, CRESCENDO predicted one binding site for the Thioredoxin protein (2trx) and 100% of the top 5 pairs of residues are within 12 Å distance (Figure 3b). To minimize the effect of noise in the plots, we used a score based on a range of subsets up to 40 positions to provide a summary score for each plot. (See Methods section for details). Using this measure, the method efficiently discriminates the correct models from the incorrect models in both these cases.

**Table 1:** Example decoy fold distribution for 3chy. Number of fold-types, strand and helix order in the fold (HI() denotes the helix order in layer I, SII() the strand order in layer II and HIII() the helix order in layer III), Number of models, Number of clusters and scores of the best model in each fold-type is detailed in this table. In the second column '-' denotes the change in the direction of the secondary structure element when compared to the native. F1 and F7 are correct folds and are in bold type.

Fold type	Strand and helix order	No. of models in each fold type in 200 Models	No. of cluster with ≤2 Å RMSD; ≥60% PID cut-off	Score of the best Model
***Native structure***	***HI (1,5);SII(2,1,3,4,5);HIII (2,3,4)***	**-**	**-**	***330.96***
**F1**	**HI(1,5);SII(2,1,3,4,5);HIII(2,3,4)**	**161**	**61**	**314.76**
F2	HI(-1,5);SII(2,-1,3,4,5);HIII(2,3,4)	3	2	202.21
F3	HI(1,5);SII(2,3,1,4,5);HIII(2,3,4)	16	11	145.19
F4	HI(1,-3,-4);SII(2,1,-3,-4,-5);HIII(-2,-5)	2	2	150.83
F5	HI(1,4);SII(2,3,1,4,-5);HIII(2,3,-5)	1	1	108.62
F6	HI(1,3,5);SII(2,1,4,3,5);HIII(2,4)	11	7	250.20
**F7**	**HI(1,5);SII(2,1,3,4,5);HIII(2,3,4)**	**5**	**4**	**260.29**
F8	HI(1,-5);SII(2,1,3,4,5);HIII(2,3,4)	1	1	67.24

The "summary score" for each plot can then be plotted against a measure of deviation from the native structure.

**Figure 4:**
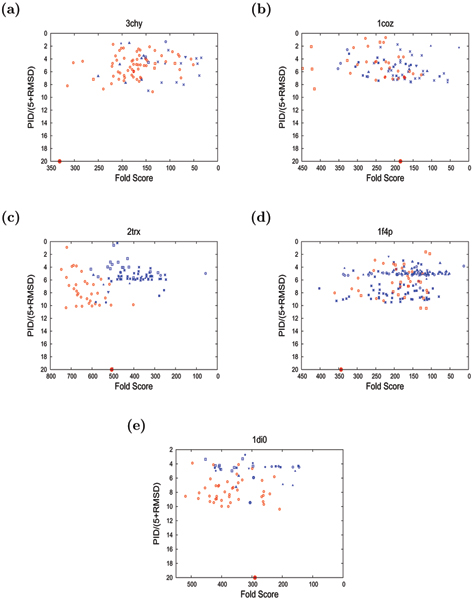
**"Summary plots" for five training-set proteins**. The "summary plots" for each of the five training-set proteins (a) 3chy, (b) 1coz, (c) 2trx, (d) 1f4p and (e) 1di0 are shown. In each plot, the "Fold Score" is plotted against the measure of structural correspondence to the native protein. (Note, both these measures are plotted on reversed scales). The best models lie towards the lower left corner in each plot having a high score and high structural similarity to the native. The native structure itself is plotted as a large red dot and all folds that correspond to the native are also red with others blue. The different symbols designate different decoy fold families.

**Figure 5:**
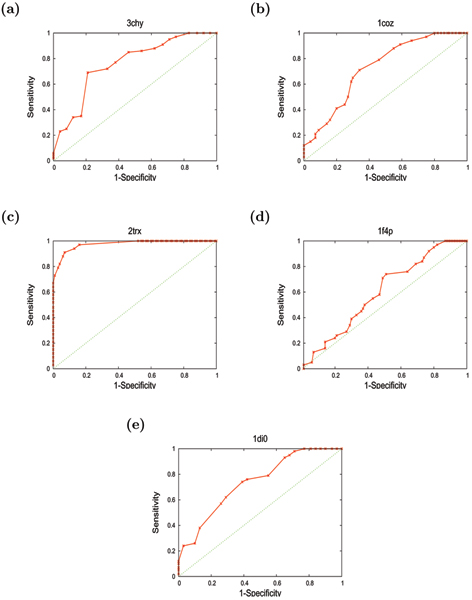
**Specificity-Sensitivity plots for five training-set proteins**. The Specificity-Sensitivity curves for each of the five training-set proteins (a) 3chy, (b) 1coz, (c) 2trx, (d) 1f4p and (e) 1di0 using "Fold Score" (red) are shown. Sensitivity = TP/(TP+FN) and Specificity = TN/(TN+FP). i.e. Specificity is defined as the fraction of significant hits (hits with scores above a threshold) being correct. Sensitivity is defined as the fraction of possible correct hits being significant. (TP = True Positives, TN = True Negatives, FP = False Positives, FN = False Negatives).

As the decoy models went through a threading phase in their construction, they can contain errors both in 3D geometry and in the register of the sequence mapped to the structure. To combine both these aspects in a single score, we plotted the percentage sequence identity (PID) of the structural superposition of the model with the native crystal structure divided by 5+RMSD (The value 5 is just a scaling factor, allowing all the folds (including the native) to be plotted with a reasonable spread). This produces plots in which each model appears as a point, allowing those with the correct fold to be compared to other decoys. (See Figures 4a to 4e, for each of the five proteins). While there is a slight trend for the correct folds to have a better RMSD and alignment (as combined in the Y-axis on Figure 4a to 4e), the important aspect of the models is whether they have the correct fold or not. With the range in structure over the decoy sets, changes in loop position can make as big a difference to RMSD as the exchange of two *β *strand positions. The latter topological difference is considered important in this work while 'random' variations in RMSD over models of same fold are not. Table 2 summarises the results shown in Figures 4a to 4e. The strength of the method in discriminating the correct and the incorrect models is also shown as ROC plots in Figures 5a to 5e for each of the five proteins. It can be seen from Figures (4 and 5) and Table 2, that proteins with the correct fold invariably score higher than those with the non-native fold. The only exception to this is a single fold (F4) from the flavodoxin set of decoys. As the flavodoxin is the largest of the five, with longer loops it might be expected to be the most likely to perform poorly. (See Methods for details about Flavodoxin fold-types).

**Table 2:** Correct and incorrect folds in top and bottom 25 ranked models. The correct folds in top 25 ranked models and the wrong folds in the bottom 25 ranked models for the five proteins are tabulated in order to show the strength of the method.

PDB	Correct in top 25 ranked models(best)	Incorrect in bottom 25 ranked models (worst)
3chy	22 (top 4)	13 (low 4)
1cozA	14 (top 4)	22 (low 11)
2trxA	24 (top 22)	25 (low 25)
1f4pA	7 (2^nd^)	23 (low 16)
1di0A	18 (top 7)	15 (low 7)

### Independent test-set of larger proteins

The method was applied to the set of seven larger *α*/*β*/*α *proteins (Figure 1). These ranged in length from 130 residues up to 187 residues thus covering a span from the previous test set up to a size well beyond any successful *de novo *or *ab initio *prediction. To evaluate success we report only the simple 1:1 RMSD value over all positions since at this size we are interested only in whether the correct fold (or a close variation) has been selected and not in the exact register of the sequence over the structure as captured by the previous measure. For each protein, the four highest ranked decoy folds were considered and the RMSD value to the native was reported. (Table 3).

**Table 3:** RMSD values for larger proteins. For each of the proteins in the larger set, the RMSD of the best model in the top four ranked fold-types is tabulated (along with the fold-type in parentheses). Where this corresponds to the native fold, the value is in bold type.

PDB (length)	Rank-1	Rank-2	Rank-3	Rank-4
1v9w (130)	13.4 (F23)	11.3 (F24)	6.9 (F26)	**6.3 (F25)**
1rlj (135)	13.7 (F10)	**4.9 (F9)**	11.2 (F1)	13.7 (F8)
1kjnA (159)	**3.4 (F1)**	5.0 (F3)	5.0 (F4)	9.4 (F6)
1vq1A (178)	8.5 (F12)	9.5 (F3)	7.1 (F5)	7.9 (F1)
1uxoA (186)	13.7 (F2)	11.4 (F14)	8.9 (F6)	11.8 (F11)
1t57A (186)	14.8 (F8)	9.8 (F10)	14.9 (F1)	9.9 (F3)
1vk2A (187)	16.4 (F13)	14.7 (F4)	14.5 (F2)	15.9 (F10)

For the smallest protein in this set, 1v9w (130 residues) the fourth ranked fold corresponds to the native with a RMSD value of 6.3 Å and the third rank model has two *β*-strands swapped between adjacent positions at the edge of the sheet. For the slightly larger 1rlj (135 residues) the second ranked fold corresponds to the native with a good RMSD value of 4.9 Å. At almost 160 residues, 1kjn was almost as good across all four top ranked models (around 5 Å RMSD) and the first ranked fold corresponds to the native. For the above three proteins, the correct fold was selected. The remaining four proteins has no native-like fold in the 200 decoy models taken from the *de novo *protein structure prediction method and so, the top ranked models were checked for them being a closer variant to the native structure. 1vq1 was considerably larger (178 residues) but for this size, the RMSD values around 7 or 8 Å on the third and fourth ranked models were acceptable. The main error in the best model was found to be caused by two adjacent *β *strands lying in swapped positions on the edge of the sheet. Indeed, neglecting strand swaps, all four models otherwise correspond to the native fold.

The remaining three proteins were close in size at 186/7 residues. At this size the two single figure RMSD values of 8.9 for 1uxo and 9.8 for 1t57 constitute good approximations to the native fold. 1t57 had a pair of swapped *β*-strand positions in the middle of the sheet and 1uxoA had a minor helix displaced to the opposite face of the sheet.

The largest protein (1vk2) had a best RMSD value over 10 Å which does not correspond to a native-like fold. The models with RMSD values 14.7 and 14.5 are not too far from the native structures. The model with RMSD 14.7, had 2 pairs of adjacent swapped *β*-strands in the middle of the sheet and a pair of helix swaps between the opposite faces of the sheet. The model with RMSD 14.5, had 2 pairs of adjacent swapped *β*-strands (one in the middle of the sheet, the other in the C-terminal end of the sheet) and a pair of helix swaps between the opposite faces of the sheet. The bigger RMSD in these models are due to longer loops and helix displacments between layers.

## Conclusion

We have shown that consideration of the requirement of proteins to form a functional site, either enzymic or binding, can be used to select the correct protein fold from a large number of well constructed decoy models. Our method uses only sequence data to do this in combination with the model structures and involves no information derived from the known structures. A test set of five small *β*/*α *type proteins were used to determine the best formulation of the CRESCENDO method, with a clear choice being indicated that the use of a reduced number of residue environments was best. This difference from the application of the method to native protein structures arises because we are applying the method to rough C*α *models from which the constructed side-chain orientations are unreliable.

This protocol was then applied to a set of larger proteins with the correct fold, or a close variant being selected in six of the seven proteins. The type of error most commonly seen in this test set was the swapping of adjacent strand positions in a *β*-sheet. With hindsight, this is not an unexpected error, since these strands still have the same orientation and any functional residues that they, or their flanking loops, carry will remain in close proximity and be scored equally well by our method. Not only will our evaluation method be blind to such variation but if elements of structure carry no functional sites then they will be equally unconstrained. The method is more sensitive to discrimination between models that have secondary structure elements oriented in the opposite direction (provided they carry the identified functional residues). For example, Chemotaxis Y protein (3chy), has the C-terminal helix (fifth) involved in binding. The fold-type F8 (Table 1), has this helix in the opposite direction, which is the lowest ranked fold for this protein. Similarly, Thioredoxin has the fourth strand involved in binding, but is in the opposite direction in one of the fold-types and ranked last. For the smaller proteins almost all loops on a face will be involved in a binding site but for the larger proteins, there is a greater chance that some loops will be unconstrained. Despite these fundamental limitations, if the number of allowed topologies can be reduced, even to single figures, then more detailed modelling methods can be applied to reconstruct the geometry of the binding site. If the nature of the substrate, or just what is bound in the site, is known then some stereo-specific constraints may provide further selection criteria.

## Methods

### Decoy model generation

Decoy models were generated from the so-called "Periodic Table" classification of protein structures [12]. These are secondary structure lattice models derived from the combinatorial enumeration of possible folds over layers of secondary structure. The lattice (or 'stick') models are converted to Cα models using a threading method and finally refined using fragments drawn from native structures [13]. When the models have the correct native fold, the RMSD against the native structure ranges from 4–6 Å in the length range of 100–150 residues (respectively). For folds that differ from the native, the RMSD ranges up to random (typically 15–20 Å in this range). Although the decoy models with the non-native fold have high RMSD values, this does not mean they are disordered. Rather they have properties (secondary structure content and packing) that are close to native proteins. In addition, some variants have only slight changes from the native fold, such as two adjacent strands in swapped position, that is often undetectable using a conventional RMSD based measure.

### Protein data

#### Chemotaxis Y protein (3chy)

This protein contains the common flavodoxin fold with 5 strands and 5 helices (2 on the I layer and 3 on the III layer) (Figure 1). The strand order in layer II is 21345, helix order in layer I is 15 and helix order in layer III is 234, which would hereafter be denoted as the following. HI(1,5);SII(2,1,3,4,5);HIII(2,3,4), where HI() denotes the helix order in layer I, SII() the strand order in layer II and HIII() the helix order in layer III. In the decoy models, eight distinct folds were found (designated F1...F8), of which F1 and F7 correspond to the native (Table 1).

#### Glycerol-3-phosphate cytidylyltransferase (1cozA)

This protein contains 5 strands and 5 helices (3 on the I layer and 2 on the III layer) (Figure 1). The strand and helix order is HI(2,1,5);SII(3,2,1,4,5);HIII(3,4). In the 200 decoy models, there were 11 different folds (F1...F11). F3 and F6 are similar to native. In the 1cozA structure the C-terminal helix packs off the sheet and this helix is a part of the active site. In fold-type F3, this helix packs on the sheet. In F6 this helix is packed off the sheet like the native crystal structure. In F5 the C-terminal helix is predicted as strand and is packed in the *β*-sheet.

#### Thioredoxin (2trxA)

This protein contains 5 strands and 4 helices (2 on the I layer and 2 on the III layer) (Figure 1). The strand and helix order is HI(2,4);SII(1,3,2,-4,5);HIII(1,3). (The negative number indicates a reverse strand direction.) In the 200 decoy models, there were 10 different folds (F1...F10). F1 is similar to native.

#### Flavodoxin (1f4pA)

This protein contains 5 strands and 5 helices (2 on the I layer and 3 on the III layer) (Figure 1). The strand and helix order is HI(1,5);SII(2,1,3,4,5);HIII(2,3,4). There were 10 different folds, among which F3 and F6 are considered as correct, although an extra strand is predicted and packed on the sheet instead of the 2nd helix which is in layer 3. The top scoring model F4 has this extra strand and strand swap between 1st, 4th and 5th strands.

#### Lumazine synthase (1dioA)

This protein contains 4 strands and 4 helices (2 on the I layer and 2 on the III layer) (Figure 1). The strand and helix order is HI(1,4);SII(2,1,3,4);HIII(2,3). In the 200 decoy models, there were 16 different folds (F1...F16). F1 is similar to native.

#### Other proteins

The method was tested with seven more 3 layer *α*/*β*/*α *proteins (1v9w, 1rlj, 1kjnA, 1vq1A, 1uxoA, 1t57A and 1vk2A). The topology diagram for the seven proteins are shown in Figure 1. The number of different topologies (fold-types) in the 200 decoy models are 29 for 1v9w, 15 for 1rlj, 14 for 1kjnA, 12 for 1vq1A, 14 for 1uxoA, 12 for 1t57A and 16 for 1vk2A.

### Clustering of models

200 models were taken from the models generated during the final step of the protein *de novo *structure prediction method [14] (see subsection 1 of Methods). Since the models are Cα models, main-chain atoms were built and side-chains were built using SCWRL [15] and refined using MODELLER [16,17]. These models consists of a variety of different folds and were classified based on their fold-types (Figure 2). Pairwise superposition between models of same fold-type was made using the program SAP [18] and the models with ≤2 Å RMSD and ≥60% PID on structural superposition were clustered together. Clustering between models of same type is needed, since the functional site prediction (when looking at the residue proximity of the predicted functional site residues) differs between models of same type due to 1) difference in loop conformation, 2) *β *strand or helix shift even by a single residue. So, even correct folds might have poor models (based on the functional site prediction). Models within each cluster were superimposed using MODELLER to get the superimposed coordinates. Average C*β *coordinates of the residues of the models of each cluster was used to find the pairwise distance between residues.

### Functional site prediction

The functional site prediction method CRESCENDO [10] was used to predict the critical residues important for binding, of the models. The environment-specific substitution tables [19] reflect the pattern of amino-acid substitutions in a particular local environment, (usually defined by local secondary structure = 4 (*α *helix, *β *strand, +phi angle and coil), solvent accessibility = 2 (accessible, inaccessible) and side-chain hydrogen bonding = 8 (side-chain:side-chain = 2; side-chain:main-chain CO group = 2; side-chain:main-chain NH group = 2), which in combination gives 64 environments i.e. 4 × 2 × 8 = 64). Restraints arising from the binding of substrates, cofactors, subunits and other molecules are not taken into account while deriving the environment-specific substitution tables. Thus, the substitution patterns of the functionally important residues are not well-predicted by the environment-specific substitution tables. So, comparison of the substitution patterns derived from the environment-specific substitution tables with the amino acid substitutions that occur during evolution in family of proteins should identify the functionally important residues (which is implemented in CRESCENDO), since they will be more conserved than that predicted from the substitution tables. The divergent score, as defined by Yona and Levitt [20], quantifies the overall difference or divergence between the observed and predicted substitution probabilities at each alignment position. The homologous sequences for the model structure is collected as described by Chelliah et al [10]. The method distinguishes residues that are conserved for a functional reason from those that are conserved for structural reason. Though the side-chains for each model were built and refined using the above mentioned programs, the hydrogen bond and accessibility information might not be accurate. Because of this, we derived the secondary structure using STICKS [21] and solvent accessibility using SACAO (K. Lin, unpublished) with the Cα models and used only 6 (3 × 2) environments. (secondary structure = 3 (*α *helix, *β *strand and coil); solvent accessibility = 2 (accessible, inaccessible).

### Fold score

Assuming that the functional residues in the correct models form clusters, and that they might be scattered in the incorrect models, a score termed the "Fold score" was calculated for each model based on the proximity of the functionally important residues. To find how the functional residues are packed in the model, pairwise distances and the product of CRESCENDO scores between each pair of residues (that are at least 8 residues apart in the linear sequence) are calculated. The resulting plot is a measure of how well the predicted site residues have co-localised in the model and will be refered to throughout as "proximity plots".

As many "proximity plots" are generated for each decoy set, we devised a summary measure of how well clustered the site residues were. The percentage of pairs of residues that are within 12 Å distance was calculated for the top 40 pairs (based on the product of CRESCENDO scores) in steps of 5, and the percentage scores were added in each step to get the final "Fold score" for that fold. The sum of the percentage of clustered residues up to a sample size of 40 was taken since, beyond this the plots generally decayed. This single statistic can then be compared to a measure of structural quality. For this we used the percentage sequence identity (PID) of the structural superposition [18] of the model with the native structure divided by the RMSD plus 5. The 5 is just a (non-linear) scaling factor that does not alter the rank of the points. PID/RMSD on a log scale would be much the same. These plots will be referred to as the "summary plots".

Specificity and Sensitivity were calculated according to the "Fold Score". Specificity is defined as the fraction of significant hits (i.e. hits with Fold scores above a threshold) being correct. Sensitivity is defined as the fraction of possible correct hits being significant.

## Competing interests

The authors declare that they have no competing interests.

## Authors' contributions

VC and WRT devised the method and wrote the manuscript together. The method was encoded and tested by VC on decoy models generated by WT.
